# Genotypic resistance testing of HCV – is there a clinical need?

**DOI:** 10.3205/id000023

**Published:** 2016-08-04

**Authors:** Andreas Walker, Rolf Kaiser, Ralf Bartenschlager, Jörg Timm

**Affiliations:** 1 Institute for Virology, Heinrich-Heine-University, University Hospital, Düsseldorf, Germany; 2 Institute of Virology, University of Cologne, Cologne, Germany; 3 Department of Infectious Diseases, Molecular Virology, Heidelberg University, Heidelberg, Germany; 4 German Center for Infection Research, Heidelberg University, Heidelberg, Germany

**Keywords:** hepatitis C, HCV, antiviral agents, resistance, viral drug resistance, direct-acting antiviral, DAA

## Abstract

Persistent infections with the hepatitis C virus (HCV) pose a profound global public health burden. In the past 5 years treatment of chronic hepatitis C has dramatically changed. Novel direct-acting antivirals (DAAs) specifically inhibiting viral enzymes or factors that are essential for the viral replication cycle have been developed and licensed for hepatitis C therapy. These novel drugs target the viral NS3/4A protease, the NS5B RNA-dependent RNA-polymerase or the replication factor NS5A. Combinations of DAAs against these targets are highly efficacious achieving virus elimination in the majority of treated patients. In countries where affordable, this rapid clinical development virtually replaced earlier interferon (IFN)-α based therapy that had been in use as standard of care for the last 25 years. With the approval of DAAs for the treatment of chronic hepatitis C the question emerged whether resistance-associated substitutions (RASs) might be of clinical relevance. Here, we discuss the available evidence for the possible benefit of resistance genotyping prior to therapy to optimize treatment of chronic hepatitis C.

## Resistance genotyping of hepatitis C virus (HCV)

With the approval of the HCV NS3/4A protease inhibitors telaprevir and boceprevir a new era of therapy of chronic hepatitis C had begun. These two drugs were the first licensed direct-acting antivirals (DAA) targeting HCV and were approved each for combination with pegylated Interferon-α (peg-IFN-α) plus ribavirin, the standard of care at that time [1], [2], [3]. Although this triple combination increased virus elimination efficiency up to ~75%, treatment options for chronic hepatitis C continued to rapidly evolve (reviewed in [4]). The most important targets for the subsequent generation of HCV-specific DAAs were the serine-type protease residing in nonstructural protein 3 (NS3) forming a complex with the NS4A cofactor, the viral replicase factor NS5A and the RNA-dependent RNA-polymerase residing in NS5B. The latter can be blocked with non-nucleosidic inhibitors or indirectly via chain termination with nucleos(t)ide analogues (reviewed in [5]). Meanwhile DAAs of all four classes have been approved and are available for treatment of chronic hepatitis C (Table 1 (Tab. 1)). Owing to high efficiency and very limited side-effects, these DAAs virtually replaced IFN-α as an essential component of HCV-specific antiviral therapy.

The goal of antiviral treatment of hepatitis C is a sustained virological response (SVR) which is defined as non-detection of HCV RNA with a sensitive assay 12 (SVR12) or 24 (SVR24) weeks after cessation of therapy [6]. Notably, all DAAs were developed with subgenomic HCV replicons, corresponding to viral “mini-genomes” that replicate autonomously in cultured human hepatoma cells. Since first generation replicons were derived from genotype 1, DAAs were optimized against this genotype and thus, had a strong bias [7]. In fact, most DAAs show highest efficacy and highest SVR rates with IFN-free treatment in patients infected with HCV genotype 1 viruses, which is the most frequent genotype in most parts of the world [8]. Broad anti-viral activity against all HCV genotypes still remains a challenge in the clinical development of DAAs and was so far only achieved by a few compounds. Therefore, most DAAs are restricted to treatment of selected genotypes [9]. Consequently, the HCV genotype continues to be an important diagnostic factor for treatment decisions and a predictor for the outcome of DAA-based therapy. Importantly, in contrast to the higher response rates of genotype 3 to IFN-treatment, the susceptibility of genotype 3 to IFN-free DAA therapy is lower compared to genotype 1 and the treatment options are limited as the currently available protease and non-nucleosidic NS5B inhibitors are not approved for genotype 3. 

With the approval of HCV-specific DAAs the question emerged whether selection of resistance-associated substitutions (RASs) might pose a clinical problem. Owing to the lack of proof-reading activity of the viral polymerase, during HCV replication mutants are continuously generated allowing rapid adaptation of the virus to altered conditions. For instance, studies of patients with acute hepatitis C observed the rapid selection of escape mutations from the host immune response, emerging within days or a few weeks (reviewed in [10]). This raised concerns that HCV might similarly adapt to drug selection pressure exerted by DAAs, thereby compromising treatment success. Indeed, in the first clinical trials with protease inhibitors in combination with peg-IFN-α and ribavi-rin it became evident that treatment failure was frequently associated with emergence of viral isolates carrying RASs that could be detected by direct sequencing of bulk PCR products amplified from virus in peripheral blood samples [11], [12]. Selection of RASs in these early therapies was enhanced in patients with poor IFN-α response, consistent with the low genetic barrier to resistance against the protease inhibitor. Consistently, patients with prior non-response to IFN-α therapy were effectively treated with a DAA monotherapy, and had a high risk of resistance selection that was associated with treatment failure [13]. In novel IFN-free treatment strategies DAAs from different classes with distinct resistance profiles were combined. Similar to the combination therapy of HIV this increases SVR rates as the drugs work synergistically and multiple substitutions in different viral targets are required for clinically relevant resistance development. Although impressive SVR rates close to 100% are achieved for different genotypes with such DAA combinations, in the few patients who experience treatment failure RASs are frequently detectable after therapy [14]. Interestingly, this is true for patients who experience a viral breakthrough under therapy as well as for patients with undetectable HCV-RNA throughout treatment, but showing a viral relapse when treatment is regularly stopped [15]. This suggests that even in the absence of detectable HCV-RNA in peripheral blood there is ongoing HCV replication below the detection limit with the possible risk of resistance selection, at least in a few individuals.

For all licensed DAAs RASs have been described *in vivo* [14], [16]. Many RASs selected in patients were previously identified in subgenomic replication models or could be retrospectively associated with a resistance phenotype when tested in such models *in vitro* [17], [18], [19]. This suggests that the available HCV cell culture models phenocopy drug resistance and are thus adequate tools to study resistance phenotypes. However, robust subgenomic replication models are available only for a few HCV subgenotypes [20] and clinical data on resistance selection in less frequent HCV subtypes are still limited. Nevertheless, the high reproducibility of the impact of key substitutions in the viral genome on the resistance phenotype in frequent HCV genotypes and subtypes allows predictions on the susceptibility to DAAs. This is the basis for interpretation tools such as geno2pheno [HCV] (publically available at http://hcv.geno2pheno.org) and allows an evaluation of viral sequences with respect to susceptibility to DAAs. The validity of the prediction strongly relies on the quantity and quality of the existing data on selected variants and the associated resistance phenotype quantified *in vitro*. Such data are currently only available for HCV subgenotypes 1a and 1b [14], [16], [21] and with some limitations for subgenotypes 3a [22] and 4d [23]. Thus, there is still substantial uncertainty about the quality of resistance phenotype prediction for less frequent HCV genotypes and subtypes.

From the clinical trials and from *in vitro* models of HCV replication it became evident that the barriers to resistance differ between DAAs. For example, first generation inhibitors of the replicase factor NS5A have a relatively low barrier to resistance whereas the barrier to resistance against the nucleotide analogue sofosbuvir is very high [24]. The S282T substitution in NS5B conferring resistance to sofosbuvir is associated with severe fitness costs for the virus [25]. Accordingly, in the absence of selection pressure variants with this substitution will replicate only very inefficiently and will be outcompeted by wildtype HCV. Thus, in sequence analyses the S282T variant appears unstable and rapidly reverts back to the parental amino acid residue probably within a few days. In contrast, high level replication is possible in case of NS5A-specific RASs [26]. Owing to limited or no fitness costs, these RASs are retained in the virus population for years and possibly infinite even in the absence of drug selection pressure suggesting that such resistant virus variants have the capacity to be transmitted and to accumulate in a population [21], [26], [27]. In line with this, the key substitution Y93H associated with high level and cross-resistance to NS5A inhibitors in all genotypes studied so far is also detectable in treatment-naïve patients [16], [28], [29]. Notably, in the absence of treatment this substitution was more frequently selected in patients carrying an IFN-λ genotype associated with more frequent spontaneous immune control suggesting that innate immune mechanisms select for this specific resistance variant [30], [31].

The barriers to resistance do not only differ between DAA classes, there are also clinically relevant differences between genotypes and subgenotypes. For example, selection of RASs in the protease domain of NS3 is more frequent in genotype 1a compared to genotype 1b [32]. In turn, selected RASs are more stable in genotype 1a and may persist for a longer time [33]. One of the key substitutions conferring resistance to multiple protease inhibitors is R155K. In genotype 1a this amino acid substitution requires only one nucleotide exchange whereas in genotype 1b exchange of two nucleotides is required. This may explain in part the lower barrier to resistance against protease inhibitors in genotype 1a compared to genotype 1b. In nearly all clinical trials testing DAA combinations with a protease inhibitor SVR rates were higher in genotype 1b than in genotype 1a supporting the notion that the difference in the resistance barrier is clinically relevant. This difference was acknowledged in the German treatment guidelines of hepatitis C [34]. For example, for patients with genotype 1a infection addition of ribavirin to the combination therapy with paritaprevir, ombitasvir and dasabuvir is recommended [35]. There is also some evidence that selection of resistance is more frequent in genotype 4d than in genotype 4a [23].

With the existing data on selection of RASs during DAA therapy of hepatitis C the question for a clinical need for resistance testing prior to therapy arose with the aim to optimize treatment decisions. The first evidence that baseline resistance testing is informative for prediction of treatment response rates emerged from trials with the protease inhibitor simeprevir. It was noted that patients carrying the substitution Q80K in HCV NS3 mounted significantly lower SVR rates when treated with a combination of simeprevir, peg-IFN-α and ribavirin [36], [37]. This Q80K polymorphism is highly prevalent in genotype 1a with reported frequencies between 15 and 30% [16], [38]. According to the approval by the European Medicines Agency (EMA) it is therefore required to exclude the Q80K substitution before simeprevir treatment of patients infected with HCV genotype 1a. Notably, in IFN-free DAA regimen with a combination of simeprevir and sofosbuvir the negative impact of the Q80K substitution on treatment response rates was only detectable in patients with cirrhosis arguing that the sofosbuvir response rate is lower in this group compared to non-cirrhotic patients, thus allowing easier selection for simeprevir resistant HCV variants. Cirrhosis is a strong negative predictive factor for treatment response in all DAA therapies so far [39], [40], [41], [42], [43]. This suggests that presence of the Q80K substitution at baseline might be more relevant as a negative predictor for treatment success when additional negative predictive factors such as cirrhosis are present. In line with this, detailed analyses of the treatment response rates according to the resistance genotype at baseline suggest that in patients with cirrhosis presence of RASs in the relevant viral targets is associated with lower treatment responses. The available data were recently summarized in a comprehensive overview by Christoph Sarrazin [16]. In a pooled analysis of patients infected with genotype 1 and treated with sofosbuvir and ledipasvir, RASs conferring high level resistance (>100 fold shift EC_50_) were associated with SVR rates of 87% compared to SVR rates ~97% in the absence of high level resistance. In a phase 3 trial with patients infected with genotype 3 HCV and treated with daclatasvir and sofosbuvir for 12 weeks, patients with liver cirrhosis showed substantially lower SVR rates compared to patients without cirrhosis [40]. Notably, the Y93H substitution in NS5A conferring high-level resistance to NS5A inhibitors was enriched in patients with cirrhosis. Accordingly, the SVR rate was also significantly lower in patients carrying the Y93H substitution compared to patients lacking the mutation at this position. Presence of the Y93H substitution in patients with advanced fibrosis may have caused insufficient efficacy of daclatasvir resulting in a factual monotherapy with sofosbuvir. From earlier trials it was already known that treatment of genotype 3 HCV with sofosbuvir plus ribavirin for 12 weeks might not be sufficient and therefore treatment with this combination for 24 weeks is recommended. This scenario reinforces the notion that baseline resistance testing is informative for predicting treatment outcome and in the case of genotype 3 infection may help to select a superior treatment strategy. 

The available information on re-treatment with a DAA combination after prior failure of a DAA therapy is still very limited [44]. There is concern that RASs may persist and become clinically relevant upon re-treatment with the same drug class. For example, in 41 patients re-treated with ledipasvir/sofosbuvir for 24 weeks after prior failure of therapy with these drugs for 8–12 weeks the SVR rate in patients carrying NS5A RASs was 60% (18 of 30) compared to 100% (11 of 11) in patients without RASs [9]. Although the numbers are still small this observation suggests that resistance genotyping may be informative for prediction of re-treatment response after failure of a DAA-based therapy and may help to identify the most effective treatment strategy.

The current recommendations of the European Association for the Study of the Liver (EASL) for treatment of chronic hepatitis C [6] do not include resistance genotyping prior to therapy. The only exception is an intended treatment of patients infected with genotype 1a with the combination of simeprevir, peg-IFN-α and ribavirin, a treatment strategy that is not frequently chosen because IFN-free options are available and have much less side effects. According to the EMA license, simeprevir is the only drug where resistance genotyping is recommended prior to treatment with this triple combination in order to exclude the Q80K substitution in HCV genotype 1a. Recently, a novel combination of the protease inhibitor grazoprevir and the NS5A inhibitor elbasvir was approved by the United States Food and Drug Administration (FDA) [45]. The current FDA license requires exclusion of NS5A RASs associated with high level resistance in patients infected with genotype 1a, because the recommended duration of therapy (12 weeks or 16 weeks) is based on the resistance genotype [45].

Given the already impressive success rates of current DAA regimens it will be a challenge to further improve SVR rates with individualized treatment strategies based on the resistance genotype. However, current treatment options pose a substantial economic burden on the public health system. Accordingly, there is also an economic need for treatment optimization including shortened treatment duration. Unfortunately, currently available data are still limited, but are necessary to allow reliable prediction of SVR rates in patients. Since the exact relevance of individual RASs is largely unclear, it will be important to distinguish the clinically important individual RASs or their combinations from reported RASs that are irrelevant for treatment decision. Moreover, in the era of ultra-deep sequencing technologies we potentially have access to detailed information on the frequencies of minor sequence variants in the viral quasispecies. In clinical studies arbitrarily chosen cut-offs have been implemented for reporting RASs at baseline, however, the clinical relevance of these cut-offs is currently discussed [46], [47]. To address these questions in the future it will be necessary to collect viral sequence data in conjunction with clinical data and treatment outcome. Importantly, information of genotypic resistance tests from clinical trials but also from patients treated outside of clinical trials is required. The test results and clinical data should be collected in large databases as only combined multi-center efforts will allow sufficiently robust analyses of the possible clinical benefits of resistance testing. Especially the few patients that experience treatment failure will be informative and resistance genotyping before and after failure should be performed.

Taken together, the clinical relevance of resistance geno-typing of HCV in the era of DAA therapy is not fully defined yet. In the opinion of the authors resistance genotyping should be performed in all patients with prior failure to DAA containing therapies. In treatment-naïve patients infected with genotype 1b viruses, currently there is not sufficient evidence available for a general recommendation of resistance testing before therapy. In contrast, there is growing evidence that resistance testing prior to therapy is informative for patients infected with genotype 1a. This has been accounted for in the current FDA license of the novel combination therapy with grazoprevir/elbasvir, but seems also reasonable prior to other combination therapies. Finally, in patients infected with genotype 3 there is strong evidence that resistance genotyping will help to optimize treatment decisions. Additional studies are clearly needed to better define the clinical need for resistance genotyping of HCV in the future.

## Notes

### Acknowledgement

This review was supported by the scientific advisory board for Antiviral Therapy of the German Association for the Control of Virus Diseases and the Society of Virology and by funds of the German Center for Infection Research (DZIF8000802-3) and the Federal Ministry of Health (IIA5-2013-2514AUK375).

### Competing interests

The authors declare that they have no competing interests.

## Figures and Tables

**Table 1:**
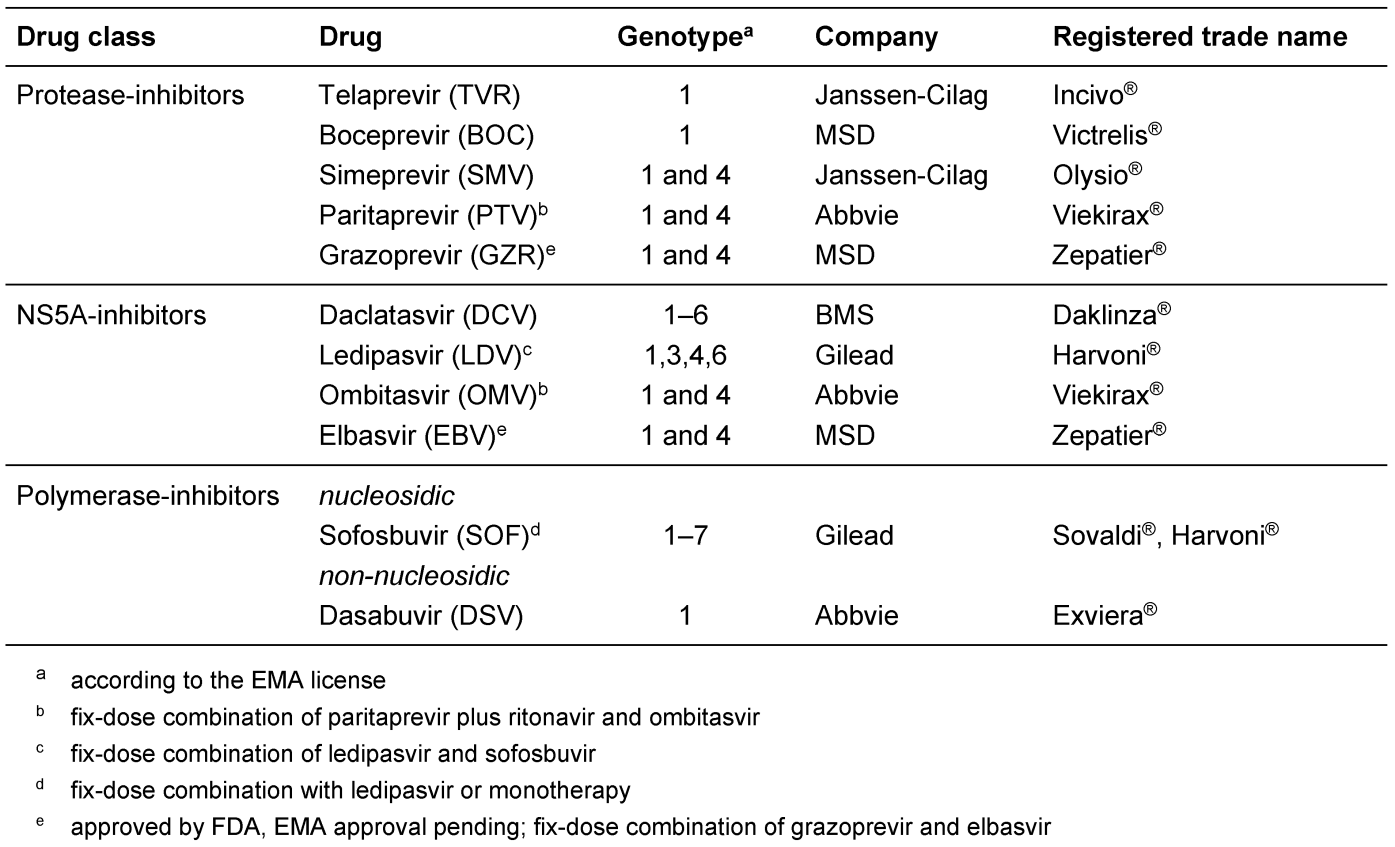
List of licensed direct-acting antivirals (DAAs) (6/2016)
